# Quantum state transfer and controlled-phase gate on one-dimensional superconducting resonators assisted by a quantum bus

**DOI:** 10.1038/srep22037

**Published:** 2016-02-24

**Authors:** Ming Hua, Ming-Jie Tao, Fu-Guo Deng

**Affiliations:** 1 Department of Physics, Applied Optics Beijing Area Major Laboratory, Beijing Normal University, Beijing 100875, China

## Abstract

We propose a quantum processor for the scalable quantum computation on microwave photons in distant one-dimensional superconducting resonators. It is composed of a common resonator *R* acting as a quantum bus and some distant resonators *r*_*j*_ coupled to the bus in different positions assisted by superconducting quantum interferometer devices (SQUID), different from previous processors. *R* is coupled to one transmon qutrit, and the coupling strengths between *r*_*j*_ and *R* can be fully tuned by the external flux through the SQUID. To show the processor can be used to achieve universal quantum computation effectively, we present a scheme to complete the high-fidelity quantum state transfer between two distant microwave-photon resonators and another one for the high-fidelity controlled-phase gate on them. By using the technique for catching and releasing the microwave photons from resonators, our processor may play an important role in quantum communication as well.

Quantum computation^1^, which can implement the famous Shor’s algorithm^2^ for integer factorization and Grover/Long algorithm^3^,^4^ for unsorted database search, has attracted much attention in recent years. There are some interesting systems which have been used to realize quantum computation, such as photons^5^,^6^, quantum dots^7^,^8^, nuclear magnetic resonance^9^,^10^,^11^, diamond nitrogen-vacancy center^12^,^13^, and cavity quantum electrodynamics (QED)^1^. Achieving quantum computation, quantum state transfer^14^,^15^ and universal quantum gates have been studied a lot, especially the two-qubit controlled-phase (c-phase) gate or its equivalent (controlled-not gate) which can be used to construct a universal quantum computation assisted by single-qubit operations^1^. To construct the high-efficiency and high-fidelity quantum state transfer and the c-phase gate on fields or atoms, cavity QED, composed of a two-energy-level atom coupled to a single-mode filed, has been studied a lot.

Simulating cavity QED, circuit QED^16^,^17^,^18^,^19^,^20^,^21^,^22^,^23^,^24^,^25^,^26^,^27^, composed of a superconducting qubit coupled to a superconducting resonator, plays an important role in quantum computation because of its good ability for the large-scale integration^28^,^29^,^30^,^31^,^32^,^33^. By far, some important tasks of quantum computation based on the superconducting qubits have been realized in experiments. For example, DiCarlo *et al.* demonstrated a c-phase gate on two transmon qubits^34^ in 2009, and they prepared and measured the entanglement on three qubits in a superconducting circuit^35^ in 2010. In 2012, Lucero *et al.*^36^ computed the prime factors with a Josephson phase qubit quantum processor and Reed *et al.*^37^ constructed a controlled-controlled phase gate to realize a three-qubit quantum error correction with superconducting circuits. In 2014, Barends *et al.*^38^ realized the single-qubit gate and the c-phase gate on adjacent Xmon qubits with high fidelities of 99.94% and 99.4%, respectively.

Interestingly, the quality factor of a one-dimensional (1D) superconducting resonator^39^ has been enhanced to 10^6^, which makes the resonator as a good carrier for quantum information processing^40^,^41^,^42^,^43^,^44^,^45^,^46^,^47^,^48^,^49^,^50^,^51^,^52^. For instance, Houck *et al.*^53^ generated single microwave photons in a circuit in 2007. In 2008, Hofheinz *et al.*^54^ generated the Fock states in a superconducting quantum circuit. In 2010, Johnson *et al.*^55^ realized the quantum non-demolition detection of single microwave photons in a resonator. In 2011, Wang *et al.*^56^ deterministically generated the entanglement of photons in two superconducting microwave resonators and Strauch *et al.*^57^ proposed a scheme to prepare the NOON state on two resonators. In 2013, Yang *et al.*^58^ presented two schemes for generating the entanglement between microwave photons and qubits. Recently, Hua *et al.*^59^ proposed some schemes to construct the universal c-phase and cc-phase gates on resonators.

There have been some theoretic studies on constructing the multi-resonator quantum entanglement and the universal quantum gate on local microwave-photon resonators in a processor composed of some resonators coupled to a superconducting qubit^57^,^58^,^59^,^60^,^61^. In this paper, we propose a quantum processor for quantum computation on distant resonators with the tunable coupling engineering^62^,^63^ between the superconducting resonator and the quantum bus. There is just one superconducting transmon qutrit *q* in our processor, which is coupled to a common resonator *R* (acts as a quantum bus). Different from the processors in previous works^57^,^58^,^59^,^60^,^61^, the resonators *r*_*j*_ (*j* = 1, 2) (act as the information carriers) in our processor are coupled to the quantum bus *R*, not the qutrit, which makes it have the capability of integrating some distant resonators^35^ by coupling them to the bus in different positions. In contrast with the resonator-zero-qubit architecture by Galiautdinov *et al.*^31^, the resonators in our processor are used for quantum information processing, not the memory elements. It does not require more superconducting qubits. With our processor, we present an effective scheme for the quantum state transfer between *r*_1_ and *r*_2_ with the Fock states 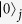 and 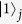 and another for the c-phase gate on two resonators by using the resonance operations between *R* and *r*_*j*_ and that between *R* and *q*. The fidelities of our quantum state transfer and c-phase gate are 99.97% and 99.66%, respectively. By catching and releasing the microwave photons from resonators^64^, our processor maybe play an important role in quantum communication.

## Results

### Quantum processor composed of resonators and a quantum bus

**Figure 1:**
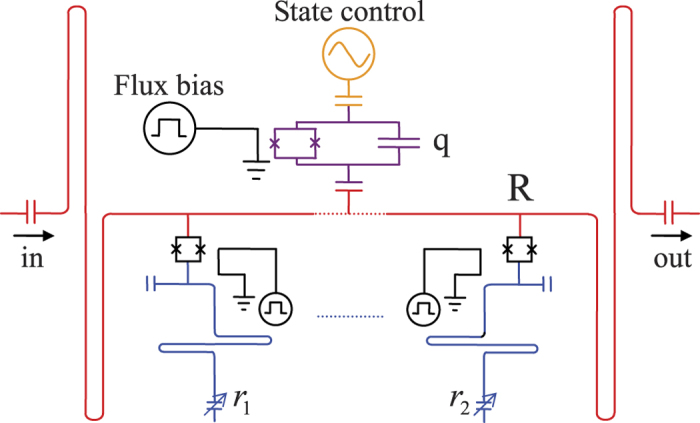
Schematic diagram for the construction of the quantum state transfer between the two microwave-photon resonators *r*_*j*_ (*j* = 1, 2) and the c-phase gate on *r*_*j*_ assisted by a quantum bus (i.e., the common resonator *R*) which is coupled to only a superconducting transmon qutrit *q*.

Our quantum processor is composed of some distant high-quality 1D superconducting resonators *r*_*j*_ and a high-quality 1D superconducting resonator *R*, shown in Fig. 1. The common resonator *R* acts as a quantum bus for quantum information processing and it is capacitively coupled to a Ξ type three-energy-level superconducting transmon qutrit *q* whose frequency can be tuned by an external magnetic field. The qutrit is placed at the maximum of the voltage standing wave of *R* (not be drawn here). The simple superconducting quantum interferometer device (SQUID) with two Josephson junctions inserted between *r*_*j*_ and *R* serves as the tunable-coupling function between them. The SQUID variables are not independent and introduce no new modes^63^. Here, the SQUIDs are not sensitive to the charge noise and can achieve a full tunability. Besides, the plasma frequencies of SQUIDs should be larger than the frequencies of the resonators. *r*_*j*_ are laid far enough to each other to avoid their direct interaction generated by mutual capacitances and mutual inductive coupling. In the interaction picture, the Hamiltonian of the processor is (*ħ* = 1, under the rotating-wave approximation)


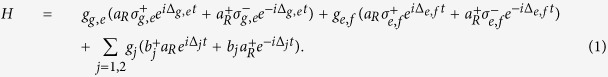


Here, 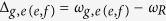 and Δ_*j*_ = *ω*_*j*_ − *ω*_*R*_. *ω*_*R*_ and *ω*_*j*_ are the the first mode frequencies of *R* and *r*_*j*_, respectively. *ω*_*g*,*e*(*e*,*f*)_ is the frequency of the transmon qutrit *q* with the transition 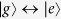 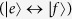 in which 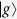, 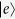, and 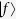 are the ground, the first excited, and the second excited states of the qutrit, respectively. 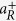 and 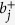 are the creation operators of *R* and *r*_*j*_, respectively. 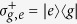 and 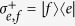 are the creation operators of the two transitions of *q*, respectively. *g*_*g*,*e*_ and *g*_*e*,*f*_ 
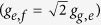
 are the coupling strengths between *R* and the two transitions of *q*, respectively. *g*_*j*_ is the coupling strength between *r*_*j*_ and *R*, which is contributed by their capacitive and inductive and can be tuned by the external flux through the SQUID^63^. By controlling the time dependence of the coupling, the cross-talk between resonators can be switched on and off.

The evolution of our processor can be described by the master equation^65^


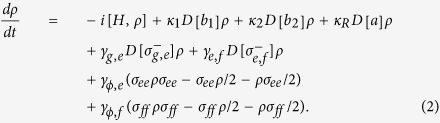


Here, the operator *D*[*L*]*ρ* = (2*LρL*^+^ − *L*^+^*Lρ* − *ρL*^+^*L*)/2 (*L* = *a*, *b*, 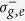, 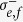). 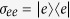 and 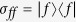. *κ*_1_, *κ*_2_, and *κ*_*R*_ are the decay rates of the resonators *r*_1_, *r*_2_, and *R*, respectively. *γ*_*g*,*e*_ (*γ*_*e*,*f*_) is the energy relaxation rate of the qutrit with the transition 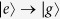 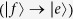. *γ*_*ϕ*,*e*_ (*γ*_*ϕ*,*f*_) is the dephasing rate of the level 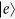 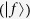 of the qutrit. To achieve the resonance operations between *R* and *r*_*j*_, the transition frequencies of all the resonators are taken equal to each other.

### Quantum state transfer between *r*_1_ and *r*_2_

Our quantum-state-transfer protocol between *r*_1_ and *r*_2_ can be completed with two resonance operations between the quantum bus *R* and the resonator *r*_*j*_. The interaction between *R* and *r*_*j*_ can be described as


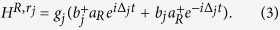


In our scheme, the states 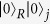, 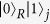, and 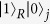 are required only. Here, the state 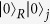 keeps unchanged with the evolution 
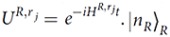
. 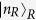 and 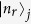 are the Fock states of *R* and *r*_*j*_, respectively. 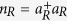 and 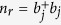. For the resonance condition between *R* and *r*_*j*_ (Δ_*j*_ = 0) and if we take the initial state of the subsystem composed of *R* and *r*_*j*_ to be 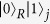, the state of the system composed of *R* and *r*_*j*_ can be expressed as (further details can be found in the method)


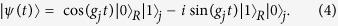


Our scheme for the quantum state transfer between the two resonators *r*_1_ and *r*_2_ can be accomplished with two-step resonance operations described in detail as follows.

Initially, we assume the initial state of the processor is


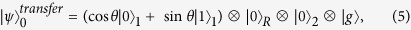


which means *r*_1_ is in the state 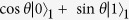, *R* and *r*_2_ are all in the vacuum state, and *q* is in the ground state. First, tuning the transition frequency of *q* to detune with *R* largely and turning off (on) the coupling strength between *R* and *r*_2_ (*r*_1_) by using the external flux through their SQUIDs, the state of the processor can evolve into


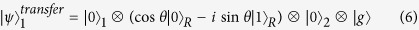


after a time of *g*_1_*t* = *π*/2.

Second, keeping the frequency of *q* detune with *R* largely, turning off *g*_1_, and turning on *g*_2_, the state of the processor can evolve from Eq. (6) to


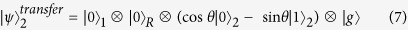


within a time of *g*_2_*t* = *π*/2. Here, we complete the quantum state transfer as


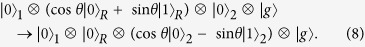


If the operation time of the second step is taken as *g*_2_*t* = 3*π*/2, the final state after the information transfer is


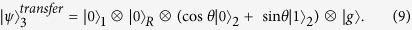


This is just the result of the quantum state transfer between the two resonators *r*_1_ and *r*_2_ from the initial state 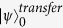.

### Controlled-phase gate on *r*_1_ and *r*_2_

C-phase gate is an important universal two-qubit gate. In the basis of two resonators 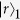 and 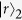 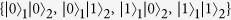, a matrix of the gate can be expressed as


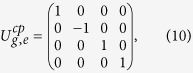


which means a minus phase should be generated if and only if the two qubits are in the state 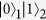. In our processor, the c-phase gate on the resonators *r*_1_ and *r*_2_ can be completed with five steps by combining the resonance operations between the quantum bus *R* and the resonator *r*_*j*_, and those between *R* and *q* with the two transitions 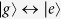 and 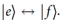


By taking the coupling strength between *q* and *R* much smaller than the anharmonicity of *q* (*g*_*g*,*e*_ ≪ *ω*_*g*,*e*_ − *ω*_*e*,*f*_), the interactions between *R* and *q* with the two transitions of 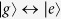 and 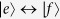 can be reduced into those of two individual two-energy-level qubits with *R*, whose Hamiltonians are


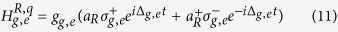


and


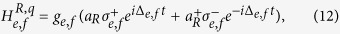


respectively. In the condition of resonance interactions between *R* and *q* with the transitions 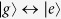 (Δ_*g*,*e*_ = 0) and 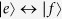 (Δ_*e*,*f*_ = 0), the time-evolution operation of the system undergoing the Hamiltonians 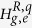 and 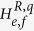 are^66^


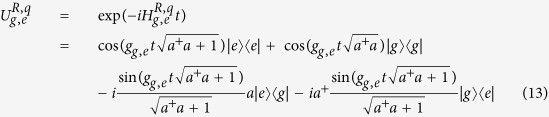


and


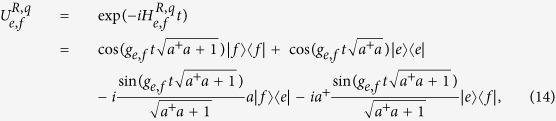


respectively.

Supposing the initial state of the processor is


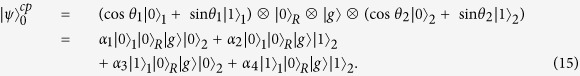


Here, the amplitudes *α*_1_ = cos*θ*_1_ cos*θ*_2_, *α*_2_ = cos*θ*_1_ sin*θ*_2_, *α*_3_ = sin*θ*_1_ cos*θ*_2_, and *α*_4_ = sin*θ*_1_ sin*θ*_2_. The five steps for the construction of our c-phase gate on *r*_1_ and *r*_2_ can be described in detail as follows.

First, turning on the coupling strength between *R* and *r*_1_ with *g*_1_ = *g*_*g*,*e*_, and turning off the interaction between *R* and *r*_2_, the state of the processor can evolve from 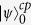 to


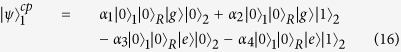


with an operation time of 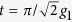
67.

Second, tuning the frequency of *q* to detune with *R* largely and turning off the coupling between *R* and *r*_1_, one can get the state of the processor as


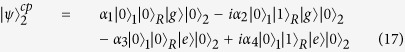


after the time of *g*_2_*t* = *π*/2 when the coupling between *R* and *r*_2_ is turned on.

Third, resonating *R* and *q* with the transition of 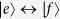 with a time of *g*_*e*,*f*_*t* = *π*, and keeping *R* uncoupled to *r*_1_ and *r*_2_, the state of the the processor becomes


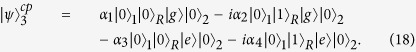


Fourth, repeating the second step, one can get the state of the processor as


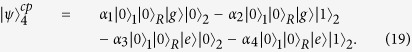


Fifth, repeating the first step, we can get the state


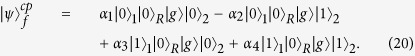


This is just the result of our c-phase gate on *r*_1_ and *r*_2_ with the initial state 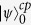.

### Possible experimental implementation

Resonance operation between a superconducting qubit and a 1D superconducting resonator has been used to achieve some basic tasks in quantum information processing, such as generating Fock states in a superconducting quantum circuit^54^, realizing the NOON state entanglement on two superconducting microwave resonators^56^, constructing the resonant quantum gates on charge qubits in circuit QED^68^ or on resonators^59^, and completing a fast scheme to generate NOON state entanglement on two resonators^69^. To get a high-fidelity resonant operation between the qubit and the resonator, the magnetic flux with fast tunability is required.

To show the performance of our schemes for quantum state transfer and the c-phase gate, we simulate their fidelities by using the whole Hamiltonian in each step. In our simulations, the parameters are chosen as: *g*_1_/(2*π*) and *g*_2_/(2*π*) can be tuned from 0 MHz to 50 MHz, *ω*_*R*_/(2*π*) = 6.65 GHz^63^, *δ* = *ω*_*g*,*e*_/(2*π*) − *ω*_*e*,*f*_/(2*π*) = 0.72 GHz^70^, g_g,e_/(2*π*) = 
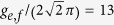
 MHz, 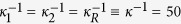 *μ*s, and 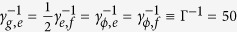 *μ*s. The transition frequency of a transmon qutrit can be tune with a range of about 2.5 GHz^71^, which is enough for us to make it detune with *R* largely. The maximal values of *g*_1_/(2*π*) and *g*_2_/(2*π*) taken by us are 50 MHz as the rotation-wave approximation can work well when the coupling strength is much smaller than the frequency of *R* and a theoretic predict of the coupling strength between two superconducting resonators can reach 1.2 GHz^63^.

The process for the generation of the initial states of 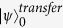 and 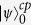 are not included in our simulations. To prepare the initial states, one should perform a proper single-qubit operation on *q* and send the information from *q* to *r*_*j*_ by using the resonance operation, the same as the one in the first step for the construction of our c-phase gate. Here, the interactions which do not attend the resonance operation should be turned off. The single-qubit operation on a superconducting qubit has been realized in experiment with a quantum error smaller than 0.0006^38^, which has little influence on our schemes. By taking the energy relaxation rate of the qutrit, the decay rates of resonators, and *g*_*g*,*e*_ and *g*_*j*_ into account, the generation of the initial states just increases a little error value of the fidelities of the quantum state transfer and the c-phase gate.

### Fidelity for our quantum state transfer

**Figure 2:**
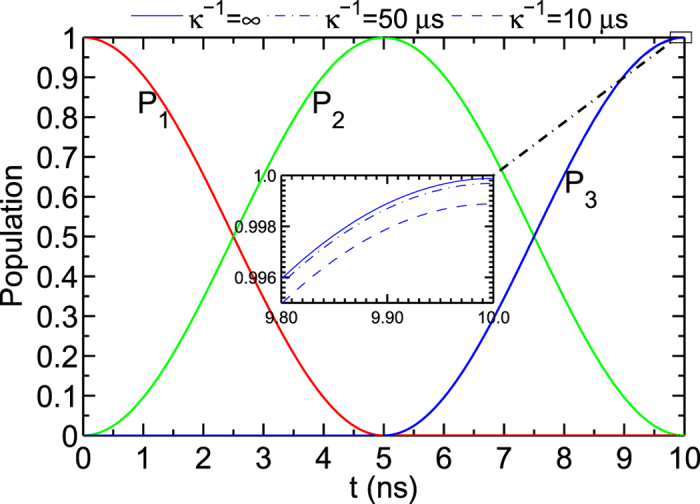
The populations of a microwave photon in *r*_1_, *R*, and *r*_2_. *P*_1_, *P*_2_, and *P*_3_ with the red, green, and blue solid lines represent the populations of the microwave photon in *r*_1_, *R*, and *r*_2_, respectively. The inset shows the populations varying with the decay rates of the resonators, in which the solid, the dot dash, and the dotted lines represent those with the decay rates of the resonators *κ*^−1^ = ∞ *μ*s, *κ*^−1^ = 50 *μ*s, and *κ*^−1^ = 10 *μ*s, respectively.

We numerically simulate the populations (vary with the operation time) of a microwave photon in *r*_1_, *R*, and *r*_2_, shown in Fig. 2. The definition of the population is


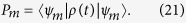


Here *m* = 1, 2, 3. 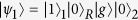, 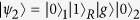, and 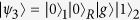. *ρ*(*t*) is the realistic density operator of the processor for the quantum state transfer from the initial state 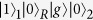. The parameters taken in the first step in our scheme are: *ω*_*g*,*e*_/(2*π*) = 5 GHz, *g*_1_/(2*π*) = 50 MHz, *g*_2_/(2*π*) = 0 MHz. In the second step, the parameters are: *g*_1_/(2*π*) = 0 MHz, *g*_2_/(2*π*) = 50 MHz, and the other parameters are the same as the ones in the first step.

From the numerical simulation, the quantum state transfer between *r*_1_ and *r*_2_ with θ = π/4 can reach a fidelity of 99.97% within 10 ns by using the definition of the fidelity as 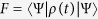 
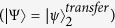
 with the initial state 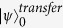. In the inset in Fig. 2, we give the three conditions of the populations with different decay rates of *r*_1_, *r*_2_, and *R*.

### Fidelity for our c-phase gate

We calculate the fidelity of our c-phase gate by using the average-gate-fidelity definition^72^,^73^


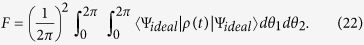


**Figure 3:**
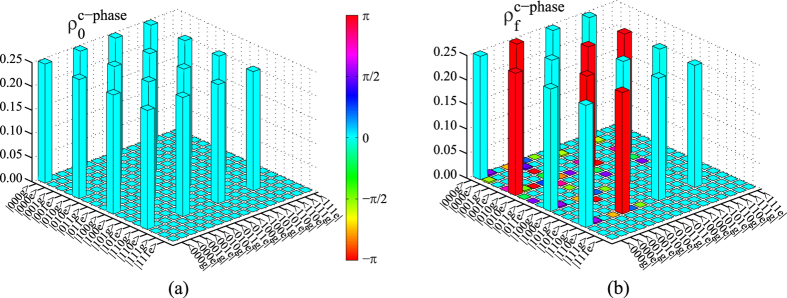
(**a**) The density operator *ρ*_0_ of the initial state 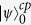 of our processor. (**b**) The realistic density operator 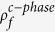 of the final state 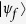 after our c-phase gate operation is performed on the two microwave-photon resonators. The color bar indicates the phase information of the density matrix elements.

Here, 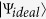 is the final state 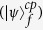 of the processor by using the ideal c-phase gate operation on the initial state 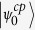. *ρ*(*t*) is the realistic density operator after our c-phase gate operation on the initial state with the Hamiltonian *H*. The fidelity of our c-phase gate reaches 99.66% within 91.5 ns by using the parameters taken in each step as shown in Table 1. Here, if we take *θ*_1_ = *θ*_2_ = *π*/4 in Eq. (15) as an example, the density operators of 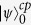 and the real final state are shown in Fig. 3(a,b), respectively.

**Figure 4:**
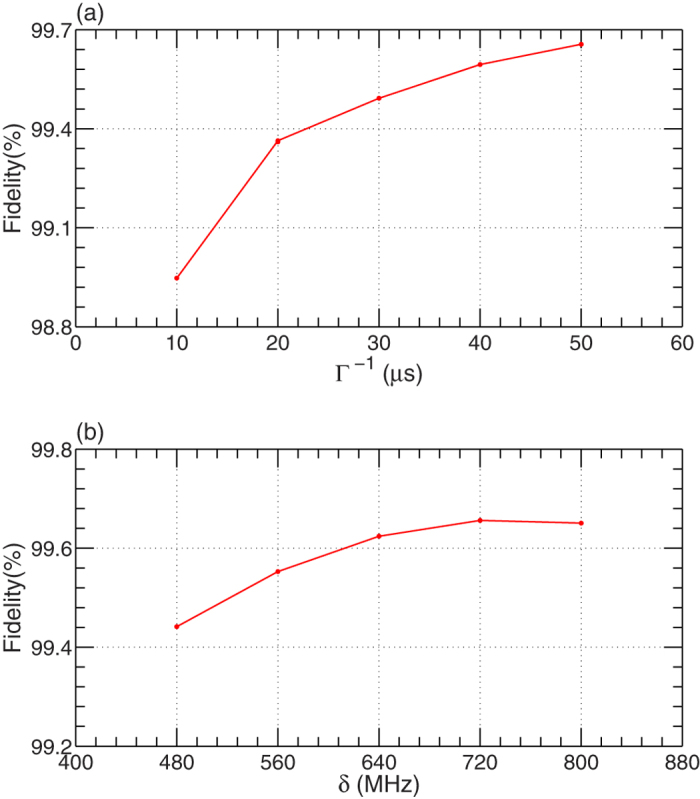
The fidelity of our c-phase gate on the two microwave-photon resonators *r*_1_ and *r*_2_ which varies with the parameters *κ*^−1^ = Γ^−1^ (**a**) and *δ* (**b**), respectively.

Actually, the fidelity of our c-phase gate is influenced by the decay rates *κ* of the resonators, the energy relaxation rate Γ of *q*, and the anharmonicity *δ* of *q*, shown in Fig. 4. In Fig. 4(a), we show the fidelity of the gate varying with the decay rates and the energy relaxation rate of the resonators and *q* (*κ* = Γ). The fidelity of the gate is numerically simulated by using different optimal parameters corresponding to different Γ (keeping *δ* = 0.72 GHz unchanged) as the competition between the operation time (leads to the error from the coherence time of the qutrit) and the coupling strength between the qutrit and the bus *R* (leads to the error from the anharmonicity of the qutrit). Here, in order to choose Γ^−1^ = 10, 20, 30, 40, and 50 *μ*s, we take 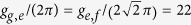, 19, 13, 13, and 13 MHz, respectively. The corresponding operation times are *t* = 58.1, 65.8, 91.5, 91.5, and 91.5 ns, respectively. By using *κ* = *ω*_*r*_/*Q* (*ω*_*r*_ is the frequency of the resonator)^16^, *κ*^−1^ = 50 *μ*s corresponds to a quality factor *Q* ~ 2.08 × 10^6^ of the resonators. In Fig. 4(b), the anharmonicity of the qutrit influences the fidelity with a small value as the coupling strength *g*_*g*,*e*_ is much smaller than *δ*, which means that the transmon qutrit in our processor does not require a large anharmonicity.

## Conclusion

To show our processor can be used for an effective quantum computation based on resonators, we have given the scheme to achieve the quantum state transfer between two resonators and the one for the c-phase gate on them. These two schemes are just based on the Fock states 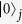 and 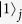 of the resonators *r*_*j*_. The fidelities of our quantum state transfer and c-phase gate reach 99.97% and 99.66% within 10 ns and 91.5 ns, respectively. In our processor, a single-qubit operation on the resonator *r*_*j*_ can be achieved with the following steps: 1), one should transfer the information from *r*_*j*_ to the qutrit with the resonance operation between them. 2), one can take the single-qubit gate on the qutrit. 3), one should transfer the information from the qutrit to *r*_*j*_. It is worth noticing that there are two steps with resonance operations in our scheme for the single-qubit operation on a microwave-photon resonator. Each resonance operation can generate a −phase for the state 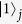 of the resonator *r*_*j*_ or the state 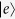 of the qutrit. The two steps with resonance operations can just eliminate this unwanted phase generated by each resonance operation as (−1)^2^ = 1. So, the single-qubit operation on the qutrit is convenient without considering the additional phase generated by the resonance operations. To readout the information of the photon states in *r*_*j*_, one can also transfer the information of the photon from *r*_*j*_ (based on the Fock states 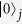 and 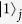) to the qutrit (based on the states 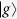 and 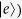 and then readout the state of the qutrit. To achieve the quantum non-demolition detection on the resonator *r*_*j*_, one can use a low-quality resonator coupled to the qutrit *q* to detect the information in the quantum bus R^55^ which comes from *r*_*j*_. By using the resonators which can catch and release the microwave photons^64^, our processor maybe play an important role in quantum communication.

In summary, we have proposed a quantum processor composed of some 1D superconducting resonators *r*_*j*_ (quantum information carriers) which are coupled to a common 1D superconducting resonator *R* (the quantum bus), not the superconducting transmon qutrit, which makes it have the capability of integrating some distant resonators for quantum information processing on microwave photons assisted by circuit QED. With this processor, we have presented a scheme for the high-fidelity state transfer between two resonators. Also, we have given a scheme for the c-phase gate on two resonators with the resonance operations. With feasible parameters in experiment, the fidelities of our two schemes are 99.97% and 99.66%, respectively. Maybe this processor can play an important role in quantum communication in future.

## Methods

### Interaction between a resonator and a qubit

In the interaction picture, the Hamiltonian of a system composed of a two-energy-level qubit coupled to a resonator (Q-R system) can be written as (under the rotating-wave approximation):


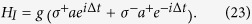


Here, *g* is the coupling strength between the qubit and the resonator. 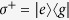 and *a*^+^ are the create operators of the qubit and the resonator, respectively. Δ = *ω*_*q*_ − *ω*_*r*_. *ω*_*q*_ (*ω*_*r*_) is the transition frequency of the qubit (resonator).

The state 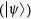 of the Q-R system can be solved with the equation of motion


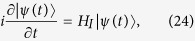


in which 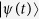 is a linear combination of the states 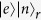 and 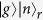, that is,


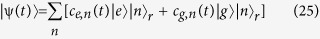


Here, *c*_*e*,*n*_(*t*) and *c*_*g*,*n*_(*t*) are the slowly varying probability amplitudes. 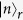 is the Fock state of the resonator. Because the only transitions between 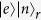 and 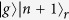 can be caused by the Hamiltonian *H*_*I*_, we just need to consider the evolutions of *c*_*e*,*n*_(*t*) and *c*_*g*,*n*+1_(*t*).

By combining Eqs. (24) and (25), one can get


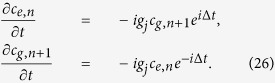


A general solution for these amplitudes is


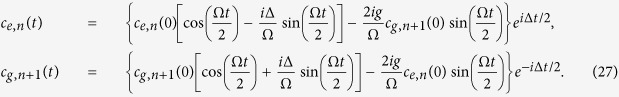


Here Ω^2^ = 4*g*^2^(*n* + 1) + Δ^2^.

## Additional Information

**How to cite this article**: Hua, M. *et al.* Quantum state transfer and controlled-phase gate on one-dimensional superconducting resonators assisted by a quantum bus. *Sci. Rep.* **6**, 22037; doi: 10.1038/srep22037 (2016).

## Figures and Tables

**Table 1:** Parameters for the construction of the c-phase gate on *r*_1_ and *r*_2_.

Step	*g*_1_(2*π*) (MHZ)	*g*_2_(2*π*) (MHZ)	*ω*_*g,e*_(2*π*) (GHz)
i	13	0	6.65
ii	0	50	5
iii	0	0	7.37
iv	0	50	5
v	13	0	6.65
